# Supplementary material for: Materials genes of heterogeneous catalysis from clean experiments and artificial intelligence
Source: MRS Bull. 2021 Oct 1;46(11):1016–26. doi: 10.1557/s43577-021-00165-6 (PMC8825435; doi:10.1557/s43577-021-00165-6)
Supplement: Supplementary file 1 — Supplementary file1 (DOCX 500 kb) [file 43577_2021_165_MOESM1_ESM.docx]

**Supporting Information**

**SISSO details**

The SISSO++ code v.0.1, was used in this work. In order to perform the $l_{0}$-optimization (sparsification) on candidate descriptors previously ranked by their correlation scores with the sure independence screening (SIS) procedure, we selected 200 candidate descriptors per dimension. These elements form the so-called SIS-selected subspaces of the total considered space of billions of candidate descriptors. These 200 candidate descriptors, in the case of dimensions higher than one, are chosen according to ten different residuals per dimension. The recently introduced^1^ multiple-residuals variant of SISSO allows the selection of descriptors with dimension $D$ based on the best $n_{res}$ descriptors with dimension $D-1$, where $n_{res}$ is the number of residuals. The use of multiple residuals enhances the diversity of descriptors identified with a fixed SIS-selected subspace size, since the best descriptor with dimension $D$ might be composed by candidate features which are present in SIS-selected subspaces obtained from diverse top-ranked descriptors of the $D-1$ dimension.

The following mathematical operators were used: addition, (absolute) difference, multiplication, division, exponential, power (2, 3 or 6), square and cubic roots, logarithm and absolute value. We set an intercept value ($c_{0}$ in eq. 4) to zero in our analysis because it provides lower CV-RMSEs compared to the situation in which this parameter is fitted (Fig. S1). This indicates a better generalizability of the resulting models when $c_{0}$ is fixed at zero.

Because the size of the descriptor candidate space grows in a combinatorial way with the number of primary features and mathematical operators, we only used part of the 50 initial primary features for the identification of descriptors with rung 3 ($q=3$). This is to keep the descriptor identification computationally feasible. For this purpose, we chose the primary features that appear in the top-ranked descriptors at the complexities of and $q=2, D=1$ for $S_{acrylic acid}$ and $q=1, D=2$ for $X_{\mathrm{propane}}$, which result in low CV-RMSEs (see Fig. S2).

For $S_{acrylic acid}$ , the primary features selected for the rung 3 analysis were:

$V_{\mathrm{fr}}^{\mathrm{pore}}$,$V_{\mathrm{act}}^{\mathrm{pore}}$,$a_{\mathrm{fr}}^{C=O}, a_{\mathrm{act}}^{C-O}$,$x_{b,act}^{V}$, $x_{s,fr}^{C}$, $x_{s,fr}^{V}$, $x_{s,act}^{V}$, $x_{s,rxn,C3}^{V}$,$x_{s,rxn,wet}^{V}$,$x_{s,rxn,dry}^{V}$, $W_{rxn,wet}$, $W_{rxn,C3}$, $\Omega_{s,rxn,C3}^{V}$, and $E_{A,act}^{\sigma}$.

For $X_{\mathrm{propane}}$, the primary features selected for the rung 3 analysis were:

$V_{\mathrm{act}}^{\mathrm{cell}}$,$s_{\mathrm{fr}}$, $V_{\mathrm{act}}^{\mathrm{pore}}$, $\lambda^{V}$, $\lambda^{O}$, $x_{s,rxn,wet}^{O}$,$x_{s,rxn,dry}^{O}$,$x_{s,rxn,C3}^{O}$, $x_{s,rxn,wet}^{V}$, $x_{s,rxn,dry}^{V}$, $W_{rxn,wet}$, $W_{rxn,dry}$,$u_{m,fr}^{O_{2}}$, $E_{A,act}^{\sigma}$, and $\tilde{\Delta\sigma}_{\mathrm{act}}^{\nu}.$

We note that for $X_{\mathrm{propane}}$, the model is sensitive when $d_{2}$ departs from the $d_{2}^{X}=\alpha^{X} d_{1}^{X}+\beta^{X}$ line, as discussed in the main text. This causes unstable predictions for such materials, limiting its applicability.

**Cross-validation details**

In order to evaluate the optimal model complexity, we performed leave-one-material-out CV. Because of the small number of materials used (nine) and the fact that some materials might be unique compared the remaining ones (e.g., MoVTeNbO_x_), the estimation of the target for the left-out material using the best model trained on the remaining materials might present abnormally low or high values (i.e., it might diverge), for example due to small denominators on the descriptor expression. This is particularly the case for higher-rung and higher-dimensional models. To circumvent this issue, we considered, for the evaluation of CV-RMSE, not only the best model, but rather the few $N_{\mathrm{ensemble}}$ best top-ranked models (ranked according to their training RMSE) when estimating the RMSE on the left-out (test) material. Within these $N_{\mathrm{ensemble}}$ models identified with the training set, we further selected the one corresponding to the lowest RMSE on the left-out material as the relevant descriptor for a given CV iteration. By doing this, we ensure a balance between the performance on the subset of data used for training and on the test material. The evolution of CV-RMSE as a function of $N_{\mathrm{ensemble}}$ (Fig. S2) indeed shows that when only the model with the best performance in the training set is considered ($N_{\mathrm{ensemble}}=1$), the CV-RMSEs might be extremely large for some models. When a few more models are considered in the ensemble, the errors drop significantly and are stabilized for ensemble sizes of ca. 25 for most of the cases. The CV-RMSE values shown in Fig. 3 and discussed in the main text correspond to a chosen $N_{\mathrm{ensemble}}=25$. We stress that the number of 25 best models analyzed is extremely small compared to the the number of descriptor candidates screened by SISSO during the descriptor identification (billions).

**Figure S1.** CV analysis for $S_{acrylic acid}$ without (A) and with (B) the fitting of the intercept for the model ($c_{0}$ in eq. 4).

**Figure S2.** Average CV-RMSE dependence on the number of top-ranked descriptors analyzed ($N_{\mathrm{ensemble}}$) for (A) $S_{acrylic acid}$ and (B) $X_{\mathrm{propane}}$.

**Figure S3.** Descriptors identified by MT-SISSO for propane conversion ($X_{\mathrm{propane}}\left( T \right)$). (A) Model expression evaluated on the nine vanadium-based catalysts of the data set at the measured temperatures (crosses), showing the quality of the fit with respect to experimental values (other markers). (B) Values of the best descriptor components for each catalyst. (C) Coefficients of the best model. The same markers and colors defining the materials in Fig. 2 are used in (A). The points in (C) are connected by splines (2^nd^ order).
